# How did Ebola information spread on twitter: broadcasting or viral spreading?

**DOI:** 10.1186/s12889-019-6747-8

**Published:** 2019-04-25

**Authors:** Hai Liang, Isaac Chun-Hai Fung, Zion Tsz Ho Tse, Jingjing Yin, Chung-Hong Chan, Laura E. Pechta, Belinda J. Smith, Rossmary D. Marquez-Lameda, Martin I. Meltzer, Keri M. Lubell, King-Wa Fu

**Affiliations:** 1 School of Journalism and Communication, The Chinese University of Hong Kong, Hong Kong, Hong Kong; 2 Journalism and Media Studies Centre, The University of Hong Kong, Hong Kong, Hong Kong; 3 Jiann-Ping Hsu College of Public Health, Georgia Southern University, Statesboro, USA; 4 School of Electrical and Computer Engineering, College of Engineering, The University of Georgia, Athens, USA; 5 Health Economics and Modeling Unit, Scientific and Program Service Branch, Division of Preparedness and Emerging Infections, National Center for Emerging and Zoonotic Infectious Diseases, Centers for Disease Control and Prevention, Atlanta, USA; 6 Research and Evaluation Team, Emergency Response Communication Branch, Division of Emergency Operations, Office of Public Health Preparedness and Response, Centers for Disease Control and Prevention, Atlanta, USA; 7 IHRC, Inc., Atlanta, USA; 8 McKing Consulting Corporation, Atlanta, USA

**Keywords:** Ebola, Social media, Network analysis, Broadcast model, Viral diffusion model

## Abstract

**Background:**

Information and emotions towards public health issues could spread widely through online social networks. Although aggregate metrics on the volume of information diffusion are available, we know little about how information spreads on online social networks. Health information could be transmitted from one to many (i.e. broadcasting) or from a chain of individual to individual (i.e. viral spreading). The aim of this study is to examine the spreading pattern of Ebola information on Twitter and identify influential users regarding Ebola messages.

**Methods:**

Our data was purchased from GNIP. We obtained all Ebola-related tweets posted globally from March 23, 2014 to May 31, 2015. We reconstructed Ebola-related retweeting paths based on Twitter content and the follower-followee relationships. Social network analysis was performed to investigate retweeting patterns. In addition to describing the diffusion structures, we classify users in the network into four categories (i.e., influential user, hidden influential user, disseminator, common user) based on following and retweeting patterns.

**Results:**

On average, 91% of the retweets were directly retweeted from the initial message. Moreover, 47.5% of the retweeting paths of the original tweets had a depth of 1 (i.e., from the seed user to its immediate followers). These observations suggested that the broadcasting was more pervasive than viral spreading. We found that influential users and hidden influential users triggered more retweets than disseminators and common users. Disseminators and common users relied more on the viral model for spreading information beyond their immediate followers via influential and hidden influential users.

**Conclusions:**

Broadcasting was the dominant mechanism of information diffusion of a major health event on Twitter. It suggests that public health communicators can work beneficially with influential and hidden influential users to get the message across, because influential and hidden influential users can reach more people that are not following the public health Twitter accounts. Although both influential users and hidden influential users can trigger many retweets, recognizing and using the hidden influential users as the source of information could potentially be a cost-effective communication strategy for public health promotion. However, challenges remain due to uncertain credibility of these hidden influential users.

**Electronic supplementary material:**

The online version of this article (10.1186/s12889-019-6747-8) contains supplementary material, which is available to authorized users.

## Background

The outbreak of Ebola in West Africa in 2014 received a disproportionate amount of media coverage and public attention relative to the threat it posed to public health in the United States [1, 2]. Mathematical models at the aggregate level have been proposed to explain the contagion process of the spread of information on social media [2]. However, a more fundamental question remains unknown—how did Ebola messages diffuse on social media platforms?

An understanding of how health information diffuses on social media is essential for public health communication. A central goal of health communication is to devise efficient and effective ways to disseminate health information [3]. In the pre-social media age, large-scale distribution of health information relied on broadcast media, such as newspaper and television. Mass media or marketing efforts rely on what might be termed a “broadcast” diffusion model, indicating that a large number of individuals receive the information directly from the same source [4].

However, Katz and Lazarsfeld [5] pointed out that interpersonal communication plays an important role in mediating information flow between mass media and the public. Because social media allows for interpersonal communication, online messages can go “viral” through a chain of individual-to-individual diffusion process, analogous to the spread of some infectious diseases. Although this “viral” diffusion model could drive large-scale diffusion to reach a large population, it is notable that the broadcast model of information diffusion still operates in social media. For example, Goel et al. [4] found that popular tweets usually spread through the “broadcast” diffusion model.

The primary purpose of this study is to examine whether the broadcast model or the viral model dominated Ebola information diffusion on Twitter. Knowing these dynamics could help public health communicators ensure messages are reaching at-risk or affected groups. Specifically, if the broadcast mechanism is dominant on social media, public health practitioners should solicit support from key opinion leaders, i.e., the most influential users, to pass on their public health messages. On the contrary, if the viral mechanism is dominant, public health practitioners should focus on the structural characteristics of individuals’ social networks (e.g. the cohesiveness of network members) [6]. In this sense, it is important to identify the influential users who can trigger large-scale information cascades, i.e., the users whose tweets were frequently retweeted. Therefore, we introduce an established method for classifying Twitter users (previously used to study non-health-related communication [7]) in order to identify influential users in the diffusion process of Ebola-related tweets.

Although previous studies have examined Twitter for its information diffusion models and the identification of influential users [4, 7], these patterns and users may vary across topics. Whether the same findings would apply to tweets related to health-related topics, such as Ebola, remains unknown. Therefore, this study aims to bridge the study of structural virality [4] and influential user identification [7] in health message diffusion. Methodologically, we propose a normalized structural virality measure as a modified version of the original measure of structural virality. Theoretically, this study extends the study of information diffusion at the aggregate level [2] to the investigation of micro-diffusion processes and the analysis of influential user types. This will advance our understanding of the differences between broadcast and viral models.

## Methods

### Data collection

Our data was purchased from GNIP, the official provider of Twitter data. We used the query “contains: ebola OR #ebola OR ébola OR #ébola” to obtain the population of Ebola-related tweets (including all retweets and replies) posted globally from March 23, 2014 to May 31, 2015 (inclusive). March 23, 2014 was chosen at the start date because it was the day when CDC began its Ebola emergency response. May 31, 2015 was the cut-off point when this data set was purchased. We obtained 36,931,362 relevant tweets, which were originated from all around the world and were publicly available. On Twitter, an original tweet is a status posted directly by the author. An original tweet can be retweeted (shared) by any other users. A retweeted status is called a retweet. The users who retweet the original tweets are retweeters. Users can follow any other users, which we call followees. Users can receive all messages posted or retweeted by their followees.

Of these relevant tweets, 52.3% (18,949,515) were original tweets. We limited our analyses to a subset of 192,209 original tweets and their retweets. Each of these 192,209 original tweets had more than 10 retweets. We excluded the less popular tweets for two reasons: first, short-lived tweets might result in isolated tweets that were not connected to and were irrelevant to the core components of a network; second, the complexity of the computational methods needed would be reduced. The 192,209 original tweets received a combined total of 12,426,623 retweets. Therefore, the combined total number of original tweets and retweets analyzed in this study was 12,618,832. The original tweets were posted by 56,768 unique handles (i.e., seed users), and the whole dataset contained 4,925,730 unique handles (i.e., users).

### Diffusion path and information cascade

**Fig. 1:**
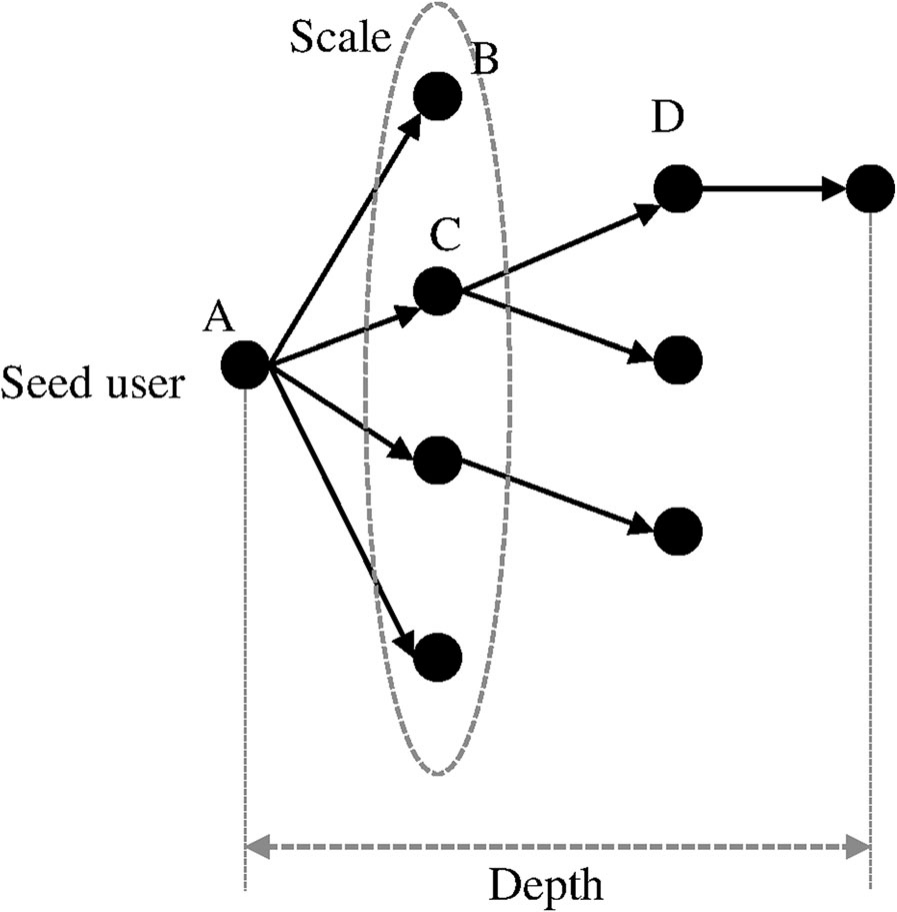
An example of information cascade and the key measures. In this example, the cascade size is 8, the scale is 4/8 = 50%, and the depth is 3

A diffusion path is the chain of retweeting that follows the posting of an original tweet. It starts with a “seed user” who sends it to their followers. For the same seed message (i.e., the original tweet), a collection of all its diffusion paths is called an *information cascade*. It can be represented graphically as a diffusion tree (Fig. 1). There are three metrics that describe an information cascade, namely cascade size, cascade scale and cascade depth (Table 1).

**Table 1:** Definition of three metrics that describe an information cascade

Metrics	Definitions
Cascade size	The number of total retweets received by an original tweet. The cascade size describes the popularity of the seed message
Cascade scale	The percentage of all retweets that were retweeted directly from the original tweet. The higher the percentage is, the more likely the diffusion cascade is dominated by the broadcast model
Cascade depth	The number of generations in a diffusion path. A large depth value may suggest a long chain of information diffusion and thus implies viral spreading

### Reconstructing diffusion paths

To determine how Ebola messages spread on Twitter, we first had to reconstruct the diffusion paths of Ebola-related messages. Information diffusion on Twitter basically depends on the “retweet” function. However, it is technically difficult to trace these paths on Twitter. First, it requires the entire population of retweets, which can only be obtained via purchase from Twitter. Second, Twitter’s official application programming interface (API) only returns the users who originally posted the tweets rather than the users from whom the retweeters directly retweeted.

**Fig. 2:**
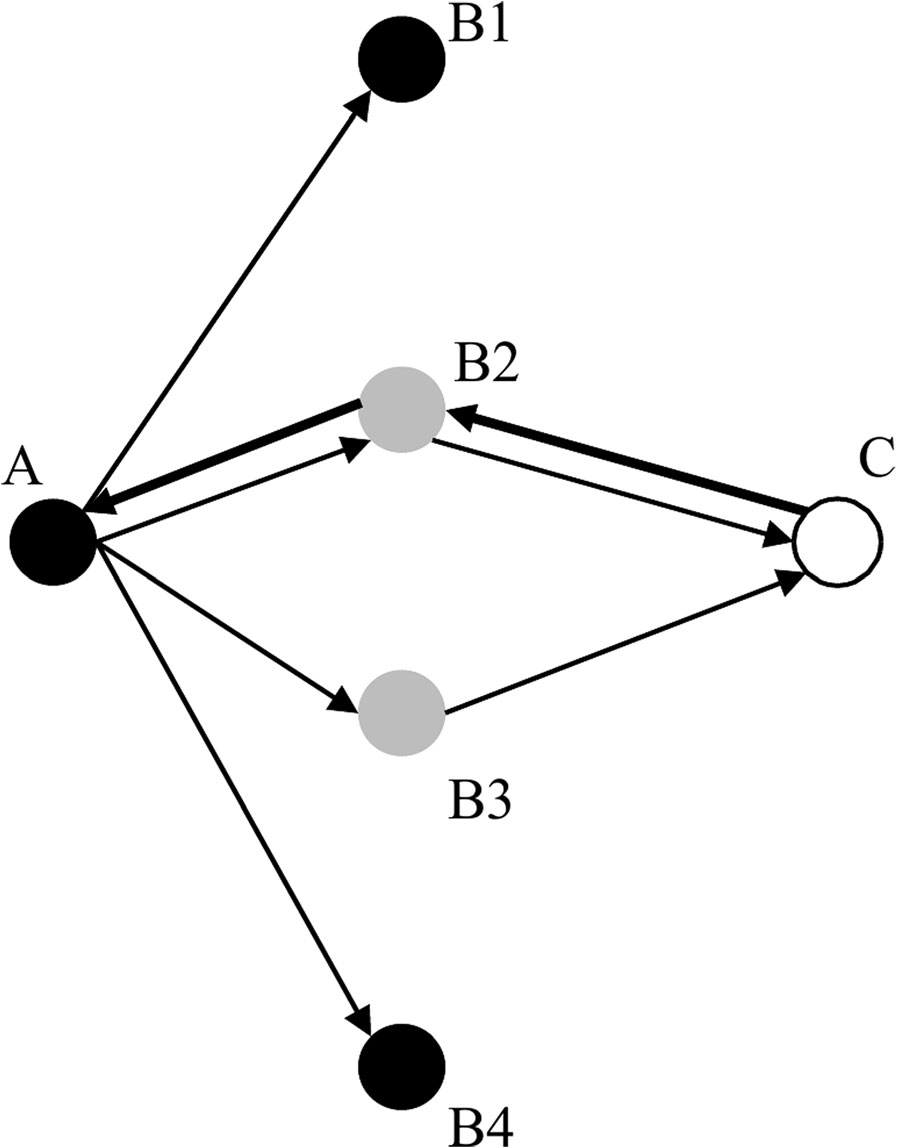
An illustration of the reconstruction of a diffusion path. From the Twitter API, we know that user A retweeted a message from user C. User A follows 4 users: B1-B4. Among the followees, users B2 and B3 follow user C and retweeted the same message from user C at time 1 and time 2 respectively. If time 1 is more recent than time 2, we will say that A retweeted C through B2 and information diffused from C to A via B2

For example, if retweeter B retweeted an original tweet posted by the seed user via retweeter A whom retweeter B followed (i.e., seed user to retweeter A to retweeter B), the Twitter API returns “seed user to retweeter B.” To solve this problem, we adopted an approach introduced in previous studies [8, 9] to reconstruct the diffusion paths. See Fig. 2 for an illustration. We reconstructed the diffusion paths of the 192,592 original tweets selected for the study.

### Measuring broadcast or viral models

The key research question of this study is to quantify the extent to which Ebola-related messages diffused through the broadcast or viral model. This was determined by calculating the structural virality and normalized structural virality for each information cascade.

*Structural virality* of a diffusion tree is defined as the average “distance” between all pairs of retweeters (known as “nodes” in network science) in the tree [4]. The distance between two nodes is the smallest number of links connecting them. In Fig. 1, the distance between A and B is 1, and the distance between B and D is 3 (B to A, A to C, and C to D). We calculated the distance between every pair of retweeters and averaged all distance values to provide a single estimate of structural virality of each diffusion tree.

The structural virality of a diffusion tree approaches a value of 2 when all retweets are directly retweeted from the seed user, which indicates that no subsequent spreading has occurred after the first generation. Structural virality reaches the maximum value when the tree is a single chain. For any information cascade, the minimum structural virality is 2 and the maximum structural virality is proportional to the cascade size (see Additional file 1). A large structural virality indicates the information cascade is likely to be a long chain and thus follows the viral model.

*Normalized structural virality*. In order to interpret structural virality more intuitively, we propose a normalized version of structural virality. We rescaled structural virality to be a normalized variable ranging from 0 (purely broadcast) to 1 (purely viral). In our analyses, we will report both the raw and normalized measures. We provide the mathematical details in Additional file 1.

### User classification

In addition to describing the diffusion structures, we identify the influential users in the information cascades. To identify influential users, we first have to develop a user classification scheme. Conventionally, influential users are measured by their authority. There are two approaches in the literature to determine authority.

The first approach is to count the number of followers a user has. In the parlance of network analysis, the authority of a user is calculated by measuring one’s degree centrality in a follower network [10] (Table 2). The underlying assumption is that users with more followers are more likely to be retweeted by others. However, this approach ignores the impact of retweets. For example, user A has 10 followers and user B has 100 followers. All 10 followers of user A retweet user A’s tweets while no follower of user B retweets user B’s tweets. If we simply use the number of followers (equivalent to the degree centrality in a follower network) as a measure of authority, we would have identified user B as more influential than user A because user B has more followers than user A. However, user A may happen to be more influential because user A’s tweets have been retweeted by all of A’s followers.

**Table 2:** Definitions of degree centrality and authority

Metrics	Definitions
Degree centrality	The total number of links of an individual in a network. In a network of followers, this will be the number of followers a user has
Authority	The relative importance of a node in a network. In this paper, we measure the authority of a user by calculating the ratio of the number of followees to the number of followers, and the ratio of the number of retweets received from others to the number of retweets the user posted

Users with more followers could be considered more influential in facilitating information diffusion. However, influence is domain specific. The first approach only accounts for follower network structure and is not informative enough to determine who is more influential in the specific context of Twitter communication pertinent to Ebola. While there is a lot of potential for information diffusion given a large number of followers, it is unclear how that potential is realized.

The second approach to determine authority is to account for the retweeting patterns in addition to the number of followers of the seed users. This approach takes into account both the potential for information diffusion offered by a follower network and the realization of such a potential for information diffusion as observed in the network pattern of retweets.

In this paper, we adopt the second approach. Following this approach, we first classify users based on their following and retweeting characteristics. Our user classification follows an established method proposed by Gonzalez-Bailon, Borge-Hothoefer and Moreno [7]. A brief explanation of the user classification method is presented in Table 3.

**Table 3:** Two dimensions of authority and definitions of four user types

First, we defined two dimensions of authority to classify users into four categories (2 × 2):	
a. Followee-follower ratio	The first dimension is the ratio of the number of followees to the number of followers. Users are classified as either ratio > 1 or ≤ 1.
b. Retweeted-retweeting ratio	The second dimension is the ratio of the number of retweets received from others to the number of retweets the user posted. Users are classified as either ratio > 1 or ≤ 1.
We expect that users, who have more followers than followees, should have more retweets by their own followers than they retweeting their followees’ tweets. Likewise, we expect that users, who have fewer followers than followees, should have fewer retweets by their own followers than they retweeting their followees’ tweets.	
Therefore, according to the two dimensions, we defined four types of users:	
a. Disseminators (also named as “Broadcasters” by Gonzalez-Bailon et al. [7]	followees ≤ followers & being retweeted ≤ retweeting
b. Common users	followees > followers & being retweeted ≤ retweeting
c. Influential users	followees ≤ followers & being retweeted > retweeting
d. Hidden influential users	followees > followers & being retweeted > retweeting

Disseminators receive fewer retweets than expected based on their number of followers. Common users received as few retweets as one would expect, given their low number of followers. Influential users received as many retweets as you would expect given their high number of followers. Hidden influential users received more retweets than expected.

In order to further explore the role of media related accounts and health organization accounts, we followed the method introduced in Towers et al. [2] to identify media related accounts. First, we compiled a list of top media organization accounts as documented in Towers et al. [2]. Second, we used the keywords such as “media” and “TV” to match Twitter’s screen names. For health organizations, we compiled a list of 65 Twitter user names, including NIH, UNICEF, UNMEER, Red Cross, WHO, and all CDC affiliated accounts.

### Statistical analysis

The unit of analysis in this study is information cascade, which is composed of retweets, except for some analyses related to user classification that are at the user level (i.e., unique Twitter handle). For the comparison between the broadcast and viral diffusion models, we plotted the probability distribution of the normalized structural virality of information cascades. We also calculated the means, medians, and standard deviations of the cascade size, cascade scale, cascade depth, and structural virality. If the cascade scale is large, and cascade depth and structural virality values are small, we can conclude that the broadcast model is dominant, vice versa. All analyses in this part were performed at the information cascade level with the number of information cascades being 192,209.

In terms of user classification, we calculated the distribution of the four user types over all users involved in the information cascades in addition to the seed users who initiated the information cascades. The unit of analysis is a unique user. That means we combined tweets and retweets posted by the same user all together.

To examine the relationships between structural virality and user types, we calculated the medians, first, and third quantiles of cascade depth, structural virality, and normalized structural virality according to different user types of the seed users. In addition, a cross-tab analysis based on the 12,426,623 retweets was performed to examine the information flow between different user types (all involved users). Since the distribution of the user types is not equal, the expected values, i.e. the number of occurrence generated purely by chance, were calculated by (column sum × row sum)/total number of cases. For example, a large number of retweets between common users is to be expected given the large number of common users in the dataset. Only when the number of retweets larger than the expected value, it indicates a significant tendency of information flow between the user types.

## Results

### Broadcast versus viral diffusion

Our analyses were based on the 192,209 information cascades of original tweets selected for the study. Given the nature of highly skewed distributions, we present both mean and median in the following section. The average cascade scale percentage in our data is high (Mean, M = 90.7%, Median, Mdn = 98.4%, Standard Deviation, SD = 15.3%). Of the 12,426,623 retweets, 91% are directly retweeted from the seed users. On average, the cascade depth of a typical diffusion tree in our data is less than 3 (M = 2.57, Mdn = 2, SD = 3.62, Max = 139). Furthermore, 47.5% of the information cascades have a depth of 1, while 70.7% have a depth of 2 or less, and 82.5% have a depth of 3 or less.

**Fig. 3:**
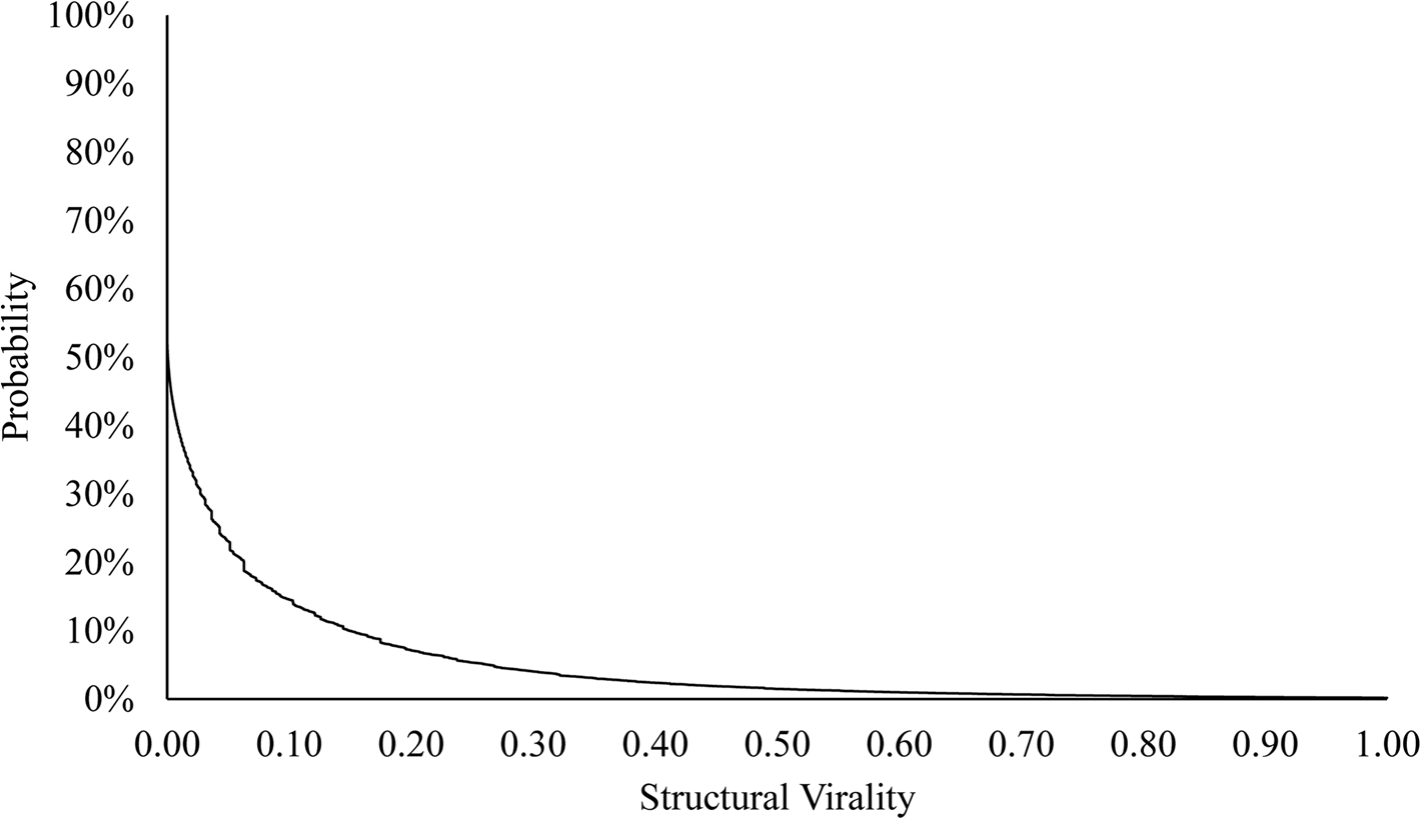
The probability distribution of normalized structural virality of information cascades of 192,209 original tweets with more than 10 retweets each, selected from a data set of 36,931,362 Ebola-related tweets from March 23, 2014 to May 31, 2015

Ebola information on Twitter spread mainly in a broadcasting pattern, given the values of the scale and depth of information cascades that we constructed from our data set. We measured how information diffused in these information cascades by using the normalized structural virality measure. Across 192,209 information cascades, the average normalized structural virality is 0.05 (Mdn = 0.0006, SD = 0.12). For the raw values, the mean is 2.27 (Mdn = 1.98, SD = 1.23). Nearly half (47.5%) of the cascades have a normalized structural virality of 0 (equivalent of having a raw value of structural virality ≈ 2), indicating a star network of retweets from the original tweet but without any further retweets. Figure 3 depicts the probability distribution of the normalized structural virality of all 192,209 cascades. The highly skewed distribution indicated that most cascades displayed broadcasting spreading diffusion, whereas only a few displayed viral spreading diffusion.

The three indicators we measured are highly correlated. First, the normalized structural virality and cascade scale are negatively correlated (Spearman’s rho = − 0.98, *p* < .01). The more structurally viral a cascade is, the less the tweet is being retweeted by multiple users at the root of the diffusion tree (for raw values, Spearman’s rho = − 0.92, *p* < .01). Second, normalized structural virality and cascade depth are positively correlated (Spearman’s rho = 0.92, *p* < .01). The more structurally viral a cascade is, the more tweets are being retweeted for multiple generations in a diffusion tree (for raw values, Spearman’s rho = 0.92, *p* < .01). Third, cascade scale percentage and cascade depth are negatively correlated (Spearman’s rho = − 0.95, p < .01). The more users retweeted the tweet at the root of the diffusion tree, the smaller is the number of generations a tweet is retweeted in a diffusion tree. Taken together, the three indicators consistently suggest that the broadcast model was dominant in the diffusion process of Ebola messages on Twitter.

Furthermore, both the broadcast model and the viral model could have generated large information cascades as the normalized structural virality and cascade size are only weakly correlated (Spearman’s rho = 0.08, *p* < .01). Among the 10 most retweeted cascades (each with more than 18,000 retweets), only two have normalized structural virality values larger than the median of 0.0006. In fact, the relationship between normalized structural virality and cascade size is non-linear: Cascades with normalized structural virality values around the median (50–60%) received the largest number of retweets on average (M = 170, Mdn = 76). The correlation between raw structural virality and cascade size is stronger (Spearman’s rho = 0.51, *p* < .01) than that between normalized structural virality and cascade size, because the average distance would be larger when there are more retweeters solely by chance.

### Identifying influential users

Number of followers ≠ influence. In the Ebola Twitter conversation, the majority of users were simply recipients and did not retweet the message; only a few users transmitted it by retweeting the message. In our data, the number of followers is moderately correlated with the number of retweets (Spearman’s rho = 0.28, *p* < .01), suggesting that equating the number of followers to influence is questionable. In fact, the most retweeted tweet in our data was posted by a user who had only 2421 followers at the time. Among the top 10 retweeted tweets, two were posted by users with fewer than 1000 followers. The average number of followers the authors of the original tweets that started the 192,209 information cascades had was 464,700 (Mdn = 30,910, and 75% of the users have more than 4077 followers).

To better measure the influence of Twitter users, we used an established method [7] that combines following and retweeting characteristics. Users who have more followers than followees are expected to have more potential to be retweeted and they are expected to be retweeted by their own followers more than they retweet others’ tweets. However, as shown in Table 4, only a small proportion of all users involved in the information cascades (2%) were retweeted as many times as expected (i.e., influential users), and the rest (38%) were retweeted less often than expected (i.e., “disseminators” as previously defined).

**Table 4:** Number of Twitter users (percentage of all users, *n* = 4,925,730) in four categories defined according to the following and retweeting characteristics of the users who tweeted about Ebola from March 23, 2014 to May 31, 2015

	One’s tweets being retweeted ≤ Retweeting others’ tweets	One’s tweets being retweeted > Retweeting others’ tweets
Followees ≤ Followers	Disseminators 1,864,885 (38%)	Influential Users 88,286 (2%)
Followees > Followers	Common Users 2,952,331 (60%)	Hidden Influential Users 20,228 (< 1%)

Note: “Followees” refers to the number of Twitter accounts that a Twitter user followed. “Followers” refers to the number of Twitter users who followed a Twitter user’s account. “One’s tweets being retweeted” refers to the number of times a Twitter user’s tweet was retweeted by others. “Retweeting others’ tweets” refers to the number of times a Twitter user retweeted another users’ tweets

Users with fewer followers than followees are generally expected to be less influential and be retweeted less often than they retweet others’ tweets. Most of such users (60% of all users) were less retweeted by their own followers as compared to how many times they retweet others’ tweets (i.e., common users). Nevertheless, a tiny proportion of users (< 1% of all users in our data set) received more retweets than they retweeted others’ tweets while they have fewer followers than followees. Thus they are categorized as “hidden influential users”.

Among the 56,768 seed users who created the information cascades, 1.7% are disseminators, 1.4% are common users, 13.7% are hidden influential users, and 83.2% are influential users. Table 5 shows that most information cascades were initiated by the influential users (91.6%), while only 1% were from common users and disseminators. The most active Twitter account was Nigeria Newsdesk (created 1657 cascades with more than 10 retweets), followed by World Health Organization (created 1309 cascades) and BBC News Africa (created 1027 cascades). All media related accounts (e.g., CNN, BBC, and New York Times) created 8.2% (15,709) information cascades and 94.7% (1068/1128) of these accounts were influential users. Nevertheless, only 2.4% of influential seed users were media related accounts. Health organization accounts created 2.1% (4080) information cascades and all the 18 health organization seed accounts were influential users. The media and health organization accounts triggered 12.8% of all retweets in our data set. In summary, although the media and health organization accounts were influential users, they accounted for only a small proportion of the cascade dynamics directly. Many other Twitter users, who served as influential users, triggered most information cascades.

**Table 5:** Cascade size, structural virality and normalized structural virality of information cascades created by four different categories of users who tweeted about Ebola from March 23, 2014 to May 31, 2015

Categories of users who created the information cascades	Percentage of total cascades	Cascade size (Q1, median, Q3)	Structural virality (Q1, median, Q3)	Normalized structural virality (Q1, median, Q3)
Influential users	91.6%	*(14, 21, 42)*	(1.89, 1.98, 2.15)	(0.00, 0.00, 0.04)
Hidden influential users	7.1%	*(13, 17, 26)*	(1.93, 2.09, 2.61)	(0.01, 0.04, 0.15)
Disseminators	0.6%	(12, 13, 16)	(1.92, 2.15, 2.64)	*(0.01, 0.09, 0.23)*
Common users	0.7%	(12, 14, 18)	(1.98, 2.28, 2.86)	*(0.04, 0.13, 0.27)*

Note: Q1: First quartile (25%); Q3: Third quartile (75%). See the User classification section in the Methods for the definition of disseminators, common users, hidden influential users, and influential users

Table 5 also presents the cascade size, structural virality and normalized structural virality of the 192,209 information cascades. Influential users and hidden influential users are more likely to trigger large cascades than disseminators and common users. We observed that both influential users and hidden influential users were likely to initiate information cascades that diffused through the broadcast model, while disseminators and common users were more likely to initiate information cascades that diffused through the viral model.

Table 6 presents the retweeting patterns among the four types of users involved in all information cascades (4,925,730 unique users and 12,426,623 retweets). The rows of Table 6 are the sources of information, while the columns are the recipients. The information flows from the rows to the columns. The values in the cells are the numbers of retweets. The expected values, indicating the number of occurrence generated purely by chance (assuming that rows and columns are independent), were calculated by (column sum × row sum)/total number of cases. For example, the value in row 1 and column 4 is 58,203, indicating that the influential users have retweeted 58,203 times from the disseminators. The observed value is larger than the expected value (shown in parentheses, 16,385), indicating that the probability of information flowing from disseminators to influential users (13.8%) is larger than the probability of information flowing at random (3.9%).

**Table 6:** Information flow, as represented by frequencies of retweets and the expected numbers in bracket, among four categories of Twitter users who tweeted about Ebola from March 23, 2014 to May 31, 2015

From-To	Disseminators	Common users	Hidden influential users	Influential users
Disseminators	*199,167* *(166,838)*	146,719 (233,779)	*19,182* *(6269)*	*58,203* *(16,385)*
Common users	82,712 (115,082)	143,088 (161,257)	*29,119* *(4324)*	*37,046* *(11,302)*
Hidden influential users	174,208 (214,220)	*306,596* *(300,172)*	*28,480* *(8049)*	*34,196* *(21,039)*
Influential users	*4,442,035* *(4,401,982)*	*6,267,010* *(6,168,205)*	107,264 (165,403)	351,598 (432,317)

Note: The numbers in parentheses are the expected values. The cells where empirical values are larger than the expected values are written in italics. The expected numbers were calculated by cross-tabulation analysis by assuming columns and rows are independent. The analysis was based on the 12,426,623 retweets

The data in Table 6 suggest that Ebola-related messages generally spread from the influential users to common users and disseminators, accounting for 86.2% (10,709,045/12,426,623) of all retweets. However, comparing to the expected values, the frequencies are somehow as expected. Another more significant route is messages flowing from common users and disseminators to influential users and hidden influential users, and then spread to the rest of the common users. This explains why the information cascades initiated by disseminators and common users have higher structural virality values (see Table 2). This is also consistent with the two-step flow theory as proposed by Katz and Lazarsfeld [5]: common users rely on the opinion leaders (i.e., the influential users or hidden influential users) to spread information widely.

## Discussion

### Principal results

Our study investigated how Ebola-related information diffused on Twitter using concepts from network analysis. We demonstrated the coexistence of two diffusion models of Ebola-related information on Twitter. The broadcast model represents one-to-many diffusion, while the viral model represents a chain of individual-to-individual diffusion. We found that the broadcast model was dominant in Ebola-related Twitter communication. Like the viral model, the broadcast model could also generate large information cascades. Furthermore, we found that influential users and hidden influential users could trigger more retweets than disseminators and common users. Disseminators and common users primarily spread information via the broadcast model. The disseminators’/common users’ tweets reached their followers, but only a small fraction of their followers retweeted them. If disseminators and common users were going to spread information beyond their immediate followers, they relied on influential and hidden influential users to retweet their tweets. If many of a disseminator’s /common user’s followers were influential or hidden influential users, then viral spreading might occur. The influential users retweeted the disseminator’s/common user’s tweets and then reached all of their followers. In this sense, it starts as a broadcast model (one-to-many) and then turns into a viral model (a chain of individual-to-individual).

Our study contributes to the existing literature in several ways. First, a previous study found that news media coverage, instead of individual-to-individual communication, dominated the dynamic patterns of Ebola-related Twitter activity in the US [2]. Our finding is consistent with their mathematical model in general – broadcast model is pervasive. However, our analysis at the micro diffusion level suggests that viral spreading still has its unique roles. Even though mainstream media and health organization accounts (such as BBC, CDC, and WHO) were very influential in terms of triggering information cascades, most influential users were not media or health organizations. They could be celebrities (e.g., Barack Obama, Bill Gates) or sports organizations (e.g., FC Barcelona). In fact, the media accounts could only account for a small proportion of all retweets in our data set. The discrepancy could be caused by the units of analysis. Towers et al.’s analyses [2] were at the aggregate level and the impact of media coverage was estimated including indirect effects. It is plausible that most of the celebrities or sports organizations in our data set actually were led by media coverage; however, the effect was not visible on Twitter. Second, our analysis was not limited to the differentiation of broadcast or viral diffusion models on Twitter. We introduced the identification of influential users [7] to extend previous studies on Ebola-related Twitter data. We found that broadcast and viral models were effective for different user types. Influential users and hidden influential users were more likely to create broadcast diffusion, whereas common users and disseminators were more likely to create viral diffusion. Finally, extending the concept of structural virality introduced by Goel et al. [4], we developed a normalized version of structural virality. The normalized structural virality will not depend on the cascade size intrinsically and can be used to analyze information cascades of all types of information across different social media platforms.

Our findings are important as they may inform how we may formulate public health communication strategy during outbreak emergency responses. If a certain type of information is more likely to diffuse via the broadcast model, it could be strategically advantageous to work with influential users and hidden influential users who can attract a large number of retweeters directly. However, if the information is more likely to spread virally, developing a successful strategy gets more complicated because viral diffusion depends on the structure of the underlying social networks. For example, information in a cohesive network – where users are well-connected with each other – spreads relatively fast [11]. One strategy for health communication would then be to identify cohesive sub-communities within a network and then spread the information in each sub-community. However, we usually do not know the whole network structure on social media platforms and therefore, the identification of sub-communities within a network may not be feasible.

Through a retrospective observational study of Ebola-related Twitter data, our analysis showed that the broadcasting model was dominant on Twitter for tweets pertinent to an emerging infectious disease outbreak, and that the broadcasting model could generate large information cascades. This finding suggests that public health practitioners may be able to rely on the broadcasting model for large-scale dissemination of public health information during outbreak emergency responses. Although it is widely believed that the viral spreading model is popular on Twitter, it is not empirically supported in our analysis of Ebola-related tweets. Viral information cascades on Twitter are rare events that public health agencies would not build communication strategies around them.

Given that the Twitter handles of many established public health agencies have more followers than followees, these Twitter handles are either “disseminators” or “influential users.” The practical question raised by health communication practitioners is how they can turn their Twitter handles from “disseminators” to “influential users” by attracting more retweets. Given the pervasiveness of the broadcasting model as observed in the retweeting patterns of Ebola-related tweets, establishing a large follower base (as did many CDC Twitter handles) appears to the most straight forward answer.

However, an outstanding question remains: how can we communicate our health messages to Twitter users who have no interest to follow public health agencies’ handles? If the broadcast model of information diffusion prevails, public health agencies’ messages would hardly ever reach these Twitter users. Our results suggest that future efforts would need to be able to identify seed users who have the ability to trigger large-scale information cascades. Our findings suggest that influential users and hidden influential users are likely to be the most important seeds. However, to collaborate with the influential users with many followers (such as celebrities) to support the cause of a specific health communication campaign may not always be the public health agencies’ priorities.

Hidden influential users would be the alternatives, as they can induce large-scale cascades beyond our expectation. However, another set of questions emerge: (a) How can we identify these hidden influential users? Can they be identified prospectively? (b) What make these Twitter users “hidden influential”? Are these users necessarily individuals or organizations with whom public health agencies should engage?

Classification of Twitter users in Table 4 is retrospective in general; however, knowledge gained from a previous outbreak may be applied to any current outbreak emergencies. However, further validations are required in future studies to ascertain user classification. The prospective identification of hidden influential users at the early stage of the communication process and the subsequent collaboration with them to propagate health messages are possible in theory but challenging in practice given the amount of work that is required to perform such analysis. The nature of the “hidden influential users” also requires our attention. Did they simply by chance write an Ebola-related tweet that became viral? Or are they individuals who are masters of online communication and can write tweets in a way that health organizations cannot? Published scholarly literature on Ebola-related Twitter data provides some insights into these highly viral tweets and who these “hidden influential users” are. Vorovchenko and colleagues [12] found that “humorous accounts” had a lot of engagement during the Ebola crisis, especially during October 2014 when Ebola cases were diagnosed in the United States. Our team’s own qualitative analysis also found that about one in four Ebola-related tweets in our dataset was either a joke or irrelevant to public health (unpublished data). Prior research on Twitter data pertinent to the 2009 H1N1 pandemic also identified humorous tweets in 8% of their sample [13]. The “hidden influential users” identified in our current study might be individuals who wrote jokes about Ebola on Twitter. These humorous tweets resonated with the emotions of many Twitter users at a juncture when many Americans were anxious about their own perceived risk of being infected with Ebola, and these tweets became viral. However, whether public health agencies should use humor in their Twitter communication to enable their tweets having a viral effect is a matter subject to debate. Given that the reputation of the government and the public health sector at large is at stake, health communicators are likely to exercise extreme caution as they approach this suggestion.

It is worth noting that the time frame of 435 days of our data surpasses many published analyses of Ebola-related tweets. As highlighted in a 2016 review, the vast majority of published Ebola-related social media studies were analyses of data from a very short time frame [14]. As described by Fung et al. and Towers et al. [1, 2], Twitter users’ attention to the West African Ebola outbreak were minimal prior to Ebola cases in the U.S. and their interest in this topic dropped off afterwards. While the cut-off point of May 31, 2015 was arbitrary (as the data was purchased in early June, 2015), our analysis encompassed the Ebola-related Twitter activities before, during and after the waves of attention to this topic that was prominent in October 2014.

### Limitations and future directions

First, the present study found that there is little difference between broadcasting and viral spreading models in terms of the number of retweets received. However, it remains unknown whether there are differences in terms of “reach” (the potential number of individuals exposed to the message), attitudes, and behavioral change. For example, some scholars claimed that interpersonal communication is more effective for behavioral change [6]. In addition, the “homophily” mechanism makes similar users gather together [15]; for example, users who follow CDC official account on Twitter (@CDCgov) may be more similar to each other than those who do not. In this way, broadcasting may reach similar users, whereas viral spreading may reach heterogeneous users across different communities on social media platforms [8]. In this sense, although broadcast model is predominant, viral spreading may be more beneficial for reaching diverse users. However, the lack of demographic data pertinent to Twitter users prevent us from further knowing the user diversity, and thereby limits the generalizability and interpretability of the findings.

Second, this is a case study of Twitter information specific to Ebola. Our findings are consistent with previous studies using general tweets [4]. However, it is unknown whether the patterns will hold across different topics. For example, does Zika-related information diffuse on Twitter differently than that of Ebola-related information [16]? Following a similar line of thought, while prior cross-sectional studies categorized contents Ebola-related tweets and manually identified Ebola misinformation [17], future research may study whether Ebola-related misinformation spreads differently on Twitter networks compared with correct scientific information. Prior study has identified a difference between the response ratio of Twitter users (the number of individuals exposed to a piece of information divided by the number of individuals taking the action to retweet it or choosing not to retweet it) for 3 news stories and 10 rumors related to Ebola [18]. In terms of prevalence, structural virality, spread, retweets, and other quantitative measures, are there any significant differences between misinformation and scientific information? A study of publicly available Facebook data found that scientific information differed from conspiracy theories in terms of cascade dynamics [19]. Addressing these issues will allow public health communicators to identify and address misinformation.

Third, even though identifying the hidden influential users to assist in the diffusion of public health messages on Twitter could potentially be more effective than encouraging influential users to share critical public health information, we employed an ad-hoc approach to identify them in the current study. Can we identify hidden influential users on Twitter (or other social media) prior to or during an emergency response? In this study, we identified many media and health organizations that were influential users. However, we also found that most of influential users were not media or health organizations. Future studies are required to find a more convenient and efficient way to identify hidden influential users.

Finally, the present study found that the broadcasting model was dominant among Ebola-related tweets. However, we do not know whether the combination of broadcasting and viral spreading strategies can facilitate the diffusion of health information beyond the additive effect.

## Conclusions

Through an analysis of a comprehensive Twitter data set, we explicitly reconstructed and described the diffusion paths of Ebola-related messages. We demonstrated that the broadcast model of one-to-many dissemination dominated the Ebola discussion on Twitter. Furthermore, we discussed the role of different user types in the diffusion process. A few influential and hidden influential users played the key role in successful diffusion of Ebola-related messages.

## Additional file

Additional file 1: Appendix Formal definition of structural virality and normalized structural virality. (DOCX 25 kb)

## Abbreviations

M: Mean
Mdn: Median
Q1: First quartile (25%)
Q3: Third quartile (75%)
SD: Standard deviation
SE: Standard error
