# Supplementary material for: How did Ebola information spread on twitter: broadcasting or viral spreading?
Source: BMC Public Health. 2019 Apr 25;19:438. doi: 10.1186/s12889-019-6747-8 (PMC6485141; doi:10.1186/s12889-019-6747-8)
Supplement: Supplementary file 1 — Appendix Formal definition of structural virality and normalized structural virality. (DOCX 25 kb) [file 12889_2019_6747_MOESM1_ESM.docx]

## Appendix

### Formal definition of structural virality

As defined by Goel et al. [4], the structural virality of an information cascade is defined as the average distance between all pairs of nodes (Twitter users in this case) in the cascade. For *n* >1 nodes,

Structural virality = $\frac{1}{n(n-1)}\sum_{i=1}^{n} \sum_{j=1}^{n} d_{ij}$

where *d_ij_* denotes the length of the shortest path between nodes (Twitter users) *i* and *j*.

### Formal definition of normalized structural virality

For any given index, if the theoretical maximum and minimum of that index are known (given well-defined conditions), we can convert into a normalized value that falls between 0 and 1, as follows,

Normalized index = (Observed value – minimum value) / (Maximum value – minimum value)

In the case of the structural virality of an information cascade, the minimum value would be obtained when the seed users’ tweet was retweeted by every retweet at the first generation but there would be no further retweets, i.e., *d_ij_* = 1 when *i* ≠ *j*, and *d_ij_* = 2 when *i* ≠ 1 and *j* ≠ 1. In other words, when the diffusion network is a star network, mathematically, the theoretical minimum value is $\frac{2\left( n-1 \right)+\frac{2\left( n-1 \right)!}{\left( n-3 \right)!}}{n(n-1)}=2-\frac{2}{n}$, given the number of nodes *n*. This value would be approximately 2, when n is large.

The maximum value would be obtained when it is the extreme viral model, when the diffusion network is a chain. The theoretical maximum value is $\sum_{k=1}^{n} \frac{\left( n-k+1 \right)\left( n-k \right)+k(k-1)}{2n(n-1)}$, given the number of nodes *n*.
